# Clonal haematopoiesis - a novel entity that modifies pathological processes in elderly

**DOI:** 10.1038/s41420-023-01590-z

**Published:** 2023-09-19

**Authors:** Ekaterina Belotserkovskaya, Vasily Golotin, Burhan Uyanik, Oleg N. Demidov

**Affiliations:** 1 Institute of Cytology RAS, 4 Tikhoretskii prospect, St. Petersburg, 194064 Russia; 2 Saint Petersburg bra­nch of “VNIRO” (“Gos­NOIRH” named after L.S. Berg), Saint Petersburg, Russia; 3 INSERM UMR1231, Laboratory of Excellence LipSTIC and label Ligue Nationale contre le Cancer, 7 Boulevard Jeanne d’Arc, Dijon, 21000 France; 4 Sirius University of Science and Technology, 1 Olimpiiskii pr-t, Sochi, 354340 Russian Federation

**Keywords:** Lymphoproliferative disorders, Senescence

## Abstract

Progress in the development of new sequencing techniques with wider accessibility and higher sensitivity of the protocol of deciphering genome particularities led to the discovery of a new phenomenon – clonal haematopoiesis. It is characterized by the presence in the bloodstream of elderly people a minor clonal population of cells with mutations in certain genes, but without any sign of disease related to the hematopoietic system. Here we will review this recent advancement in the field of clonal haematopoiesis and how it may affect the disease’s development in old age.

## Facts

- Clonal haematopoiesis is a condition affecting elderly people.
- Clonal haematopoiesis is associated with mutations in certain genes that give expansion advantages to the cells.
- Mutations associated with clonal haematopoiesis change the functions of immune cells.
- Clonal haematopoiesis may affect various diseases and their response to treatment.

## Open Questions

- Is there any difference in clonal haematopoiesis with mutations in epigenetic regulators versus DNA damage response genes?
- What are the consequences of clonal haematopoiesis to immune system?
- Can subdivide Clonal haematopoiesis into several subtypes based on mutation profile and prognosis?

## Introduction

New high-throughput sequencing techniques were widely introduced into clinical practice at the beginning of the XXI century. In contrast to the classical Sanger sequencing protocol, next-generation sequencing (NGS) can detect mutations, even if they are present only in a small number of cells of investigated sample [1]. In order to detect a mutation by classical Sanger sequencing, ~20% of the cells in the tissue sample must carry this mutation in their genome [2]. Then new generation sequencing allows us to determine the mutation when the mutated allele is found in 1% of cells and less [2]. The advantages of next-generation sequencing greatly expanded our knowledge of the spectrum of mutations found in tumors, and confirmed the theory of tumor heterogeneity when tumor cells with mutations on different parts of the mutation spectrum were detected in the same tumor [3]. Blood is one of the most accessible and most frequently studied samples for clinical diagnosis. It is not surprising that, following the whole genome analysis of tumor tissue, a large amount of data has been accumulated from the early 2000s from the NGS analysis of blood cells. Bioinformatic analysis of these data showed that in the blood of both healthy people and patients with various diseases, there are clones of cells carrying a particular mutation. The presence of such clones with mutations was strictly correlated with the age of the subject [4–10]. In the young age group (<45 years) mutations were found in <1% of the cases [6, 10]. In elderly people over 60 years old, the phenomenon of clonal haematopoiesis was detected in 10% of people and more [6, 8, 10]. Thus, clonal haematopoiesis (CH) is appearance of hematopoietic cells with certain mutations detected in at least 1% of blood cells [11]. The term “clonal haematopoiesis of indeterminate potential” (CHIP) was first introduced by David Steensma and Benjamin Ebert in 2015 for individuals carrying somatic leukemia-associated mutations at variant allele frequency (VAF) ≥ 2% [11]. Since 2015, more than 100 articles have been published that describe this phenomenon.

It can lead to changes in the functions of the gene and its products, but does not significantly affect the morphology of cells and does not cause any pathological condition in the hematopoietic system immediately. That was a reason why CH was also called Clonal Haematopoiesis of Indeterminate Potential (CHIP) in contrast to the clonal detection of cells already associated with morphological changes (Myelodysplastic syndrome, MDS) or disease (Acute myeloid leukemia, AML). Despite the last statement, with time the clones of cells with mutations can serve as a reservoir for the emergence of new additional mutations and the gradual onset of a preleukemic state (MDS), leukemia [6, 8, 10], lymphoma [12–14], and multiple myeloma [15, 16]. CHIP increases the risk of developing leukemia, but most of the patients with CHIP will never develop malignancies. It should be noted that mutations in certain genes could change the function of blood cells, which in turn can affect the course of concomitant diseases, including cardiovascular diseases [17–19].

## The spectrum of genes with mutations in clonal haematopoiesis

Modern sequencing methods open a possibility to detect mutations in one or several genes contained in a small number of cells in the analyzed sample, and screen large groups of people simultaneously. In a study by Giulio Genovese et al. [6], the whole-genome sequencing of peripheral blood cells from more than 12 thousand individuals was performed. These people were not specifically pre-selected for cancer or hematological abnormalities. As expected, signs of clonal haematopoiesis were detected in 10% of persons over 65 years of age and only in 1% of people under the age of 50 [6]. A list of the genes frequently mutated in clonal haematopoiesis is given in the Table 1. It is interesting to note that when a similar study was performed on a group of cancer patients without any sign of onco-hematological pathology, the frequency of occurrence of genes with mutations changed. The genes involved in cellular response to DNA damage, such as *PPM1D*, *TP53*, and *ATM*, were found to be among the most frequently mutated genes [20]. This frequency bias to DNA-damage response genes can be explained by sampling, that included therapy-related patients.

**Table 1:** Top genes frequently mutated in CHIP.

Gene name	Functions	Prevalent type of mutations	Consequences of mutations	Mutation frequency, %			Implication in clonal haematopoiesis-related disorders
				CHIP	De novo MDS	De novo AML	
Epigenetic modifications							
* DNMT3A*	Methyltransferase catalyzing genome-wide DNA methylation de novo [91, 92]	Missense [93]	Loss-of-function: complete or partial loss of the catalytic function of the DNMT3A methyltransferase or impairment of interactions with other proteins [29] resulting in increased self-renewal activity of HSCs [94]	29–56 [10, 21, 22, 63, 69]	2.6–20 [95–99]	18–23 [25, 100]	- Atherosclerosis [18, 73, 101, 102] - Heart failure with ischemic and nonischemic disease [70] - Degenerative aortic valve stenosis [17] - Сhronic obstructive pulmonary disease [4, 69] - Coronary heart disease [18] - Myocardial infarction (TET2) [18] - Infectious diseases [80] - Myeloid neoplasms [97, 103, 104]
* TET2*	Methylcytosine dioxygenase converting 5-methylcytosine into 5-hydroxymethylcytosine [105, 106], that is essential for the normal development of HSCs [107]	Missense, nonsense, and frameshift [5]	Loss-of-function mutations associated with DNA hypermethylation of enhancers, including those of tumor suppressor genes and thus inducting leukemogenesis [108]	15–27 [10, 21, 22, 63, 69]	19–26 [27, 109–111]	6–27 [112–114]	
* ASXL1*	Polycomb protein participating in histone modification of chromatin and thus regulating polycomb-mediated repression of genes involved in cell proliferation [115]	Frameshift/nonsense in the last exon [116]	The effect of mutations are still controversial: loss-of-function (truncated protein is associated with modulation of methylation in H3K27 histone, impairing normal haematopoiesis) [116] or gain-of-function (via HOX genes upregulation) [117–120]	3.5–11 [10, 21, 22, 63, 69]	14.4–19 [99, 110]	5–17 [26, 121, 122]	- Atherosclerosis [18] - Chronic ischemic heart failure [64] - Coronary heart disease [18] - Myocardial infarction [18] - Infectious diseases [80] - Myeloid neoplasms [104, 123]
DNA damage response							
* PPM1D*	Phosphatase involved in dephosphorylation and inactivation of DNA damage response pathways [124, 125]	Nonsense or frameshift in 5–6 exons [31, 32]	Gain-of-function mutation is characterized by truncated protein with enhanced stability and activity [31]	2.5–8 [6, 10, 21, 22, 69]	3 [126]	1.2 [127]	- Therapy-related myeloid neoplasms [23]
* TP53*	Tumor suppressor transcription factor involved in cell stress and DNA damage response [128]	Missense [6, 8, 10]	Gain-of-function mutations lead to interact p53 with EZH2 and enhances its association with the chromatin, thereby increasing the levels of H3K27me3 in genes regulating HSPC self-renewal and differentiation [129]	2–8 [10, 21, 22, 69]	7.5–9.4 [110, 130]	7–18 [41, 93, 131, 132]	- Therapy-related myeloid neoplasms [9, 104, 133, 134]
Cell signaling							
* JAK2*	Tyrosine kinase involved in hematopoietic growth factor signaling	Missense [135]	Gain-of-function mutation leads to enhanced the JAK2 kinase activity and constitutive growth signaling [136]	0.1–10 [4, 137–139]	3–5 [110, 140]	0.5–5.2 [141–146]	- Thrombosis [147, 148] - Myocardial infarction [18] - Atherosclerosis [18, 149] - Myeloid neoplasms [104]

The most frequently mutated gene associated with CHIP in the majority of works on clonal haematopoiesis is *DNMT3A* [8, 10, 20–22]. The second place is usually shared by two other epigenetic regulators, *TET2* and *ASXL1* genes [8, 10, 21, 22]. The fourth place in a number of works is given to the *PPM1D* serine-threonine phosphatase genes [8, 10, 21, 22]. It should be mentioned that mutations in the *PPM1D* gene are often detected in individuals with a history of chemotherapeutic drug treatment [23]. Mutations in the *DNMT3A*, *ASXL1*, and *TET2* genes can contribute to the development of blood cancer, and are frequently found in MDS [24] and AML patients [25–28]. It is assumed that oncogenic transformation is associated with impaired epigenetic regulation of the entire genome, for example, impairment of DNA methylation in the case of *DNMT3A* mutations, the DNA methyltransferase gene [29].

**Fig. 1:**
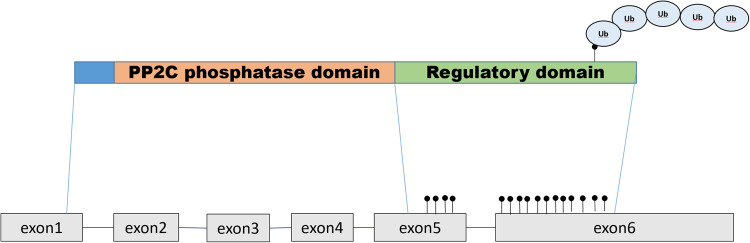
Various mutations, shift of frame or deletion in the *PPM1D* gene are shortening the expressed protein and preventing expression of regulatory domain of phosphatase located in the fifth and sixth exons. This leads to the disappearance of polyubiquitation (Ub) site, which is a signal for proteasome protein degradation. Thus, the protein is stabilized and present in the cell at higher concentration than normal.

Mutations in the *PPM1D* gene have previously been detected mainly in nonhematopoietic tumors [30]. The majority of *PPM1D* mutations in clonal haematopoiesis are located in the last two exons [6], which leads to the loss of the regulatory domain of the product of this gene, serine-threonine phosphatase Wip1 [31]. This leads to elevated levels of the enzyme in the cells due to protein stabilization [31]. Figure 1 shows that various mutations, shift of frame or deletion, are shortening the expressed protein and preventing the expression of the regulatory domain of phosphatase located in the fifth and sixth exons. This leads to the disappearance of the polyubiquitation (Ub) site, which is a signal for proteasome protein degradation [32]. Thus, the protein is stabilized and present in the cell at a higher concentration than normal. Despite the fact that Wip1 is a regulator of the activity of the tumor suppressor p53 [32], this type of mutation does not have a significant correlation with the hematological cancers. The presence of genetic amplifications of *PPM1D* was shown by us and others in 2002 [33, 34] Interestingly, in 2008 a group led by Sakaguchi K. published a splice form PPM1D430, which is almost identical to the shortened gene products produced by mutations found in clonal haematopoiesis [30]. L. Makurek’s group first described the “gain-of-function” mutations in the sixth exon of *PPM1D* in tumors [31].

Mutations in the *PPM1D* gene in CHIP not only result from chemotherapy, but also provide resistance to chemotherapy, which contributes to the expansion of a clone with an increased level of the gene product, Wip1 phosphatase [23]. We have also shown the increased resistance of cells with an increased level of Wip1 to a combination of chemotherapeutic drugs, oxaliplatin and 5-fluorouracil [35].

The origin of CHIP in the haematopoietic stem cell compartment and the influence of clonal mutations on the differentiation of mutated hematopoietic stem cells (HSCs) into mature blood lineages are still to a large extent obscure. Using VAF analysis of 91 mutations in six peripheral blood cell fractions of CHIP carriers, significantly higher VAFs were found in monocytes, granulocytes, and NK cells compared to B and T lymphocytes. Thus, these data indicate a predominant involvement of monocytes, granulocytes, and NK cells in CHIP [36]. Besides that, investigation of lineage repartition patterns in peripheral blood and bone marrow samples from individuals with CHIP revealed mutated Lin-CD34 + CD38- hematopoietic stem cells as cells of CHIP origin [36]. It is also worthy of note interesting findings concerning to *DNMT3A* mutation frequency. The *DNMT3A*-carriers demonstrated higher T-cell VAFs compared other analyzed CHIP genes. It was suggested that *DNMT3A* mutations could be earlier event in HSC affection or played minor role in myeloid bias [36]. In line with this investigation, VAF analysis of different immune cell lineages in another group of CHIP carriers showed the highest prevalence of *DNMT3A* mutation lesions in the haematopoietic multipotent cell compartment, rather than *TET2* mutations being dominant in the myeloid cell lineage [37]. These findings suggest a distinct role of *DNMT3A* and *TET2* mutational lesions in the differentiation pathway of affected HSC. Another similar study on the role of *ASXL1* mutational lesions revealed that *ASXL1* mutations are more specific for myeloid-primed progenitors or involved in myeloid bias [38]. Thus, DTA (*DNMT3A*, *TET2*, *ASXL1*) mutations are thought to play different roles in mutant HSC differentiation, with *TET2* and *ASXL1* lesions being responsible for myeloid bias.

## Possible immunological consequences of clonal haematopoiesis

Franceschi C et al. [39] proposed a mathematical model of “inflammatory aging” (inflammoaging) and suggested that the expansion of immune cell clones with mutations resulting from spontaneous clonal haematopoiesis in old age is responsible for the creation of a pro-inflammatory microenvironment in the organs and tissues of an aging organism, which is part of the aging of the immune system - immunosenescence. Thus, one of the possible consequences of clonal haematopoiesis is the appearance of immune cells with altered properties that can affect the functioning of the immune system.

**Fig. 2:**
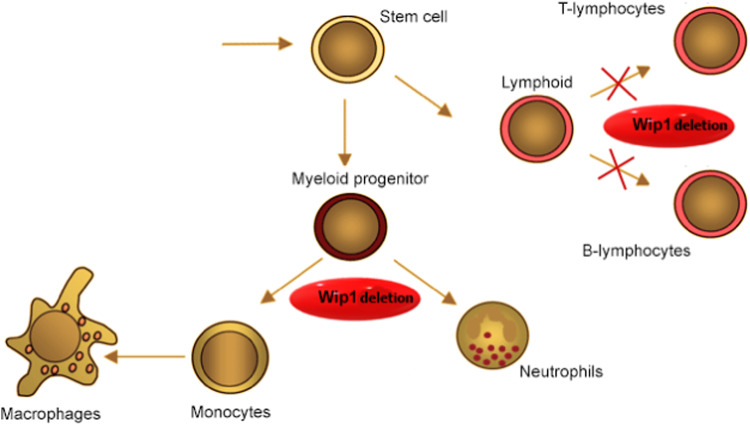
The PPM1D role in immune system and cell differentiation. A deletion in the *PPM1D* gene promotes the differentiation of myeloid cells into monocytes and neutrophils, and also inhibits the differentiation of lymphoid into T and B lymphocytes.

For example, as mentioned above, mutations associated with CHIP occur in 5–6 exons of the *PPM1D* gene and lead to the stabilization of the protein [32]. These changes lead to increased levels of the serine-threonine phosphatase protein, Wip1, which plays a significant role in important signaling pathways in immune cells (Fig. 2).

The role of PPM1D in the cells of the immune system was studied mainly on *PPM1D* knockout (KO) mice [40, 41]. We and others have shown that the Wip1 encoding gene, *PPM1D*, is expressed in hematopoietic progenitors, HSCs and all immune cell lines including neutrophils, macrophages, B and T lymphocytes in both bone marrow and peripheral blood [42], and plays an essential role in several physiological pathways [43, 44].

PPM1D has been shown to promote the active proliferation of HSCs [45]. Normally, elevated levels of PPM1D are observed in blood, intestinal, and mesenchymal stem cells [46]. During aging its amount decreases. Decreased level of *PPM1D* expression leads to the faster aging of stem cells and an increased occurrence of apoptosis [47, 48]. Wip1 plays an essential role in the fate of these stem cells [47, 48], particularly in the hematopoietic compartment where it has been shown that Wip1 modulates HSCs functional activity and differentiation through mTOR pathway [45].

Notably, PI3K/AKT/mTOR pathway is an important target of PPM1D signaling [49]. In several models, it has been described how Wip1 modulates this signalization [49–51]. Indeed, activating phosphorylation of mTOR at Ser2448, 2481, and 2159 and phosphorylation of mTOR downstream target, p70S6 Kinase, are dephosphorylated by Wip1 in a direct or ATM-dependent manner [49, 50]. Besides inhibition of mTOR signaling pathway, Wip1 is involved in reducing AKT and PI3K signaling by dephosphorylation of AKT and inhibition of Rac1-GTPase in an ATM-dependent and independent manner [52–54].

In physiological conditions, Wip1 activity is dedicated to the maintenance of HSCs quiescence and the facilitation of their differentiation [45]. Wip1 deficiency leads to the premature aging phenotype of HSCs, which is associated with higher self-proliferation rates and a poorer capability to differentiate [45].

Given the role played by Wip1 in normal haematopoiesis, the *PPM1D* mutations that can occur during CHIP can disturb the production and amount of circulating immune cells. Moreover, Wip1 cannot only modulate HSC compartment – it is also known to regulate the differentiation and functional activity of several immune effector cells [55, 56].

Wip1 plays an essential role in the development of one of the most important effectors of immune response, T cells [55, 57]. Wip1 positively regulates T cell development at several levels.

Using *Ppm1d*-deficient mouse models, Choi et al. [40] described an absence of proliferative responses of T cells in Wip1^-/-^ mice. This phenomenon is partially explained by the necessity of Wip1 activity during the maturation of T cells inside the thymus. Briefly, the development of T effectors answers to a well determined spatial and temporal differentiation program which begins with the entry of CD4- CD8- double negative lymphoid progenitors into the thymus, which then progress through 4 main stages of development (DN1 to DN4) to become CD4+CD8+ double positive. They then undergo a re-arrangement of their TCR, and then a positive or negative selection during the transition to simple positive T lymphocyte. Schito et al. [55] determined that the block of T-cells development at the DN3 stage in *Ppm1d*-KO mice led to reduced numbers of DP thymocytes which where prone to apoptosis, subject to abnormal cell cycles, and associated with the reduced size of lymphoid organs. Using Wip1^-/-^ and p53^-/-^ double KO mouse models, they showed that Wip1 controls cell death and cell cycle arrest at the DN3 stage of T-cell development in a p53-dependent manner. Moreover, Sun et al. [57] reported a few years later, that Wip1 is a critical regulator of the functional thymic stroma. Wip1 modulates in an intrinsic manner medullary thymic epithelial cell maturation through negative regulation of the p38MAPK pathway. Therefore, Wip1 is essential to the normal development of T lymphocytes and the maintenance of the functional organization of the thymus, a key organ of the immune compartment, by preventing the hyperactivation of the p53 and p38MAPK pathways.

Another essential component of adaptive immunity has also been shown to be positively regulated by Wip1. Similarly, to T-cell differentiation, the differentiation of the common lymphoid progenitor to B cells is known to be associated with mechanisms that generate elevated levels of DNA damage and p53 activation. In this context, Yi et al. [56], showed that Wip1 is able to promote B-cell maturation and proliferation by keeping in check the p53-mediated pro-apoptotic pathway.

The importance of Wip1 activity for T effector cells and B cells, two major components of adaptive immunity, underline even more the potential negative consequences of the *PPM1D* mutations during CHIP on the efficiency of the immune response.

The significance of Wip1 in the immune compartment does not only limit to adaptive immunity. Indeed, it has not only been shown that Wip1 controls myeloid lineage differentiation, but that it can also modulate inflammatory response [40, 58–60].

Several groups have described how Wip1 increased activity during the maturation and production of neutrophils, thus preventing the differentiation of common myeloid progenitors (CMPs) to pro-inflammatory mature granulocytes, to the detriment of other myeloid lineages [61]. Under normal conditions, the inhibition of the p38MAPK-STAT1 pathway by Wip1 is essential to prevent neutrophilia and the normal development of myeloid lineages [61]. Wip1’s influence is not limited only to myeloid cell differentiation; Sun et al. [57] described that phosphatase Wip1 is an intrinsic negative regulator of many pro-inflammatory cytokines and seems especially important for the control of migration and pro-inflammatory behavior of neutrophils through the negative modulation of NFKB, p38 MAPK, and STAT1 pathways.

Therefore, the hyperactivation of Wip1 by the *PPM1D* mutations that can appear during CHIP could lead to a defective immune response through the inhibition of previously cited pathways and an absence of inflammatory response, which is necessary for coordination and good activity of immune system.

Ultimately, these various functions of PPM1D in the immune system indicate that immune response could be highly affected by clones of cells bearing the *PPM1D* mutations during CHIP.

## The effect of clonal haemopoiesis on diseases of the cardiovascular system

The presence of the mutations described above in blood cells can have a significant effect on the cardiovascular system and can alter the course of diseases. Clonal haematopoiesis leads not only to blood cancer, but also to diseases of the cardiovascular system [8, 17–19, 62], autoimmune diseases [63], and also reduces life expectancy [8, 18, 19, 64, 65]. The number of abnormalities in the genome of blood cells increases with age [22, 66]. Comparison of blood stem cells from old and young mice showed that older animals had high prevalence of clonal hematopoiesis in the bone marrow. It indicates an increased mutation rate [67].

CHIP correlates with increased mortality [8, 64]. Surprisingly, in CHIP carries after 80 years of age clonal haematopoiesis is not a factor of higher risk of death [68, 69] while many studies have reported, that younger individuals with CHIP are characterized by inferior survival [6, 8, 22]. This suggests that clonal haematopoiesis affects all body systems. Indeed, the association of clonal haematopoiesis with the risk of developing cardiovascular diseases, in particular atherosclerosis, was shown [8, 18, 62]. CHIP-carries has been proved to characterize higher risk of ischemic stroke, heart failure, and myocardial infarction in contrast to patients without clonal haematopoiesis [18, 62, 64, 70]. It is important to emphasize that the development of cardiovascular diseases can be initiated by mutations associated with blood cancer (*DNMT3A*, *JAK2*, *ASXL1*, and *TET2*) [18, 21, 64]. In patients harboring these mutations, the vessels were more calcified, which is a sign of developing atherosclerosis [18]. This was probably due to dysfunctions of macrophages, which, in the presence of a *TET2* mutations, express an increased level of cytokines and chemokines (for example, interleukin 1β, IL-6, IL-8) [62, 71, 72]. In turn, this leads to inflammation and the formation of atherosclerotic plaques. It was shown that in mouse models of atherosclerosis the size of the plaques increased significantly after the transplantation of bone marrow cells with mutations in the *TET2* gene [73]. Contrary to traditional understanding, it has been recently suggested atherosclerosis is a cause of CHIP [74]. This study reported, that atherosclerosis conditions promoted higher hematopoietic stem cell division rate, that in turn facilitated CHIP emergency [74]. Suggested hypothesis was confirmed in mouse models of atherosclerosis. Although molecular determinants of atherosclerosis that promote HSC proliferation are unclear [75], undoubtedly this model is of great interest.

**Fig. 3:**
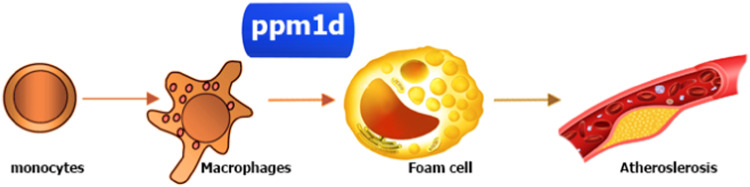
The role of PPM1D in atherosclerosis. PPM1D accumulation increases the number of foam cells which ultimately contributes to the development of atherosclerotic plaques in blood vessels.

Our laboratory primarily focused on studying the functions of the *PPM1D* gene. We have established the role of this gene in inflammatory diseases and in oncogenesis [47, 52, 76, 77]. In addition to the importance of *PPM1D* in oncology, it was found that *PPM1D* played an important role in atherosclerosis, and its role was realized by regulating the formation of “foam” cells of atherosclerotic plaque [50] (Fig. 3). Due to the frequent occurrence of *PPM1D* mutations in clonal haematopoiesis [23, 78] and the involvement of the gene in the regulation of immune functions [42] it is promising to study the effect of immune cell alterations on the course of cardiovascular diseases.

## Clonal haematopoiesis and other disorders

Although, most studies about relationship CHIP with disorders are devoted to blood neoplasms and CVDs, more and more findings concerning other diseases has been reported. Some studies have demonstrated, that CHIP is associated with chronic obstructive pulmonary disease (COPD) [4, 69], which is accompanied with inflammatory state.

Notably, recent studies revealed associations of CHIP with infection diseases [79, 80]. It has been shown CHIP is a risk factor for bacterial (Clostridium Difficile, Streptococcus/Enterococcus) [79] and viral infections (human immunodeficiency virus (HIV)) [80]. Furthermore, potential association between CHIP and severe Covid-19 outcomes is a current subject of debate. Although published data dealing with CHIP as a risk factor for Covid-19 patients are controversial [79, 81–83] it is important to emphasize, that CHIP and severe Covid-19 have a number of common features, both are typical for elderly people, being associated with cardiovascular and neoplasm disorders, proinflammatory conditions [79]. Proinflammatory state as connection between Covid-19 and CHIP is under intensive examination. Recently it has been shown that CHIP-carries are characterized by higher IL-6 in serum [72] and C-reactive protein [84], conditions are also similar to Covid-19 [85, 86]. The molecular mechanisms linking Covid-19 and clonal haematopoiesis as well as opportunity to use CHIP analysis as a biomarker of severe Covid-19 are the subject of ongoing research.

## Conclusion

Clonal haematopoiesis is defined not as a pre-leukemic condition, such as myelodysplastic syndrome, but as a condition with an undefined potential in which mutations that appear in cells can lead, or not lead, to the development of the disease. This depends on a number of factors, including the appearance de novo new genetic mutations in other genes, which in most cases does not occur and clonal haematopoiesis a priori does not end with a pathological condition. Therefore, clonal haematopoiesis is a potential pre-pathological condition that could have an effect on immune, cardiovascular, and other systems and organs. It has to be considered during the development of an individual protocol for the diagnosis, treatment, and rehabilitation of patients with various diseases.

The study of the immunological consequences of clonal haematopoiesis is necessary for a clearer understanding of the immune system in the elderly, since this phenomenon is observed mainly in aging people. The aging of the population is a modern global trend and one of the main challenges to biomedical science due to the increase in the number of patients aged 60 years and more, and due to an insufficient level of accumulated knowledge in the field of physiology and pathology of aging, including the field of immune system aging.

The term clonal haematopoiesis with undetermined potential (CHIP) was introduced initially to describe processes that contribute to leukemogenesis. Today, it becomes obvious that these mutations instead increase the resistance of hematopoietic cells to various stresses, including genotoxic stress during chemotherapy. Therefore, CHIP in most of the cases is a protective reaction of the organism. Moreover, these mutations do not definitely lead to leukemia.

It is important to pay attention, that clonal haematopoiesis has been found to contribute in early screening of solid cancer using non-invasive blood test of tumor-derived mutations in plasma samples, so-called cell-free DNA analysis (cfDNA) or cancer liquid biopsy. Nowadays plasma cell-free DNA analysis is used as clinical tool for early cancer diagnostics, therapy response monitoring, and minimal residual disease detection [87]. However, recent studies have shown tumor-derived mutations in plasma cell-free DNA included clonal haematopoiesis-related mutations [87–90]. Presence of clonal haematopoiesis-related mutations in cell-free DNA is challenge for interpretation of cell-free DNA test. To overcome this issue matched plasma and white blood cells sequencing is recommended [87]. However, this distinction is especially problematic in case of similar mutations for both solid tumor and clonal haematopoiesis, like *TP53* abnormalities. For this reason, cfDNA test in advance-aged patients and patients exposed chemotherapy needs to be added with diagnostics for CHIP-related mutations in blood.

Interestingly, CHIP with mutations in the *PPM1D* gene, without causing hematologic changes leads to such changes in the hematopoietic system that promote oncogenesis. This is probably done by creating a pro-tumor environment [78]. In further studies of clonal haematopoiesis, we should pay attention to the functional changes in the immune system introduced by CHIP mutations, since these changes can affect not only oncogenesis, but also modify the role of immune cells in the course of various diseases.

## Data Availability

Data sharing not applicable to this article as no datasets were generated or analyzed during the current study.

## References

[1] Arsenic R, Treue D, Lehmann A, Hummel M, Dietel M, Denkert C (2015). Comparison of targeted next-generation sequencing and Sanger sequencing for the detection of PIK3CA mutations in breast cancer. BMC Clin Pathol.

[2] Rohlin A, Wernersson J, Engwall Y, Wiklund L, Björk J, Nordling M (2009). Parallel sequencing used in detection of mosaic mutations: comparison with four diagnostic DNA screening techniques. Hum Mutat.

[3] Choi S, Chu J, Kim B, Ha SY, Kim ST, Lee J (2019). Tumor heterogeneity index to detect human epidermal growth factor receptor 2 amplification by next-generation sequencing: a direct comparison study with immunohistochemistry. J Mol Diagnostics.

[4] Buscarlet M, Provost S, Zada YF, Barhdadi A, Bourgoin V, Lépine G (2017). DNMT3A and TET2 dominate clonal hematopoiesis and demonstrate benign phenotypes and different genetic predispositions. Blood.

[5] Busque L, Patel JP, Figueroa ME, Vasanthakumar A, Provost S, Hamilou Z (2012). Recurrent somatic TET2 mutations in normal elderly individuals with clonal hematopoiesis. Nat Genet.

[6] Genovese G, Kähler AK, Handsaker RE, Lindberg J, Rose SA, Bakhoum SF (2014). Clonal hematopoiesis and blood-cancer risk inferred from blood DNA sequence. N Engl J Med.

[7] Gillis NK, Ball M, Zhang Q, Ma Z, Zhao Y, Yoder SJ (2017). Clonal haemopoiesis and therapy-related myeloid malignancies in elderly patients: a proof-of-concept, case-control study. Lancet Oncol.

[8] Jaiswal S, Fontanillas P, Flannick J, Manning A, Grauman PV, Mar BG (2014). Age-related clonal hematopoiesis associated with adverse outcomes. N Engl J Med.

[9] Takahashi K, Wang F, Kantarjian H, Doss D, Khanna K, Thompson E (2017). Preleukaemic clonal haemopoiesis and risk of therapy-related myeloid neoplasms: a case-control study. Lancet Oncol.

[10] Xie M, Lu C, Wang J, McLellan MD, Johnson KJ, Wendl MC (2014). Age-related mutations associated with clonal hematopoietic expansion and malignancies. Nat Med.

[11] Steensma DP, Bejar R, Jaiswal S, Lindsley RC, Sekeres MA, Hasserjian RP (2015). Clonal hematopoiesis of indeterminate potential and its distinction from myelodysplastic syndromes. Blood.

[12] Gibson CJ, Lindsley RC, Tchekmedyian V, Mar BG, Shi J, Jaiswal S (2017). Clonal hematopoiesis associated with adverse outcomes after autologous stem-cell transplantation for lymphoma. J Clin Oncol.

[13] Husby S, Favero F, Nielsen C, Sørensen BS, Bæch J, Grell K (2020). Clinical impact of clonal hematopoiesis in patients with lymphoma undergoing ASCT: a national population-based cohort study. Leukemia.

[14] Venanzi A, Marra A, Schiavoni G, Milner SG, Limongello R, Santi A (2021). Dissecting clonal hematopoiesis in tissues of patients with classic hodgkin lymphoma. Blood Cancer Discov.

[15] Chitre S, Stölzel F, Cuthill K, Streetly M, Graham C, Dill C (2018). Clonal hematopoiesis in patients with multiple myeloma undergoing autologous stem cell transplantation. Leukemia.

[16] Mouhieddine TH, Sperling AS, Redd R, Park J, Leventhal M, Gibson CJ (2020). Clonal hematopoiesis is associated with adverse outcomes in multiple myeloma patients undergoing transplant. Nat Commun.

[17] Mas-Peiro S, Hoffmann J, Fichtlscherer S, Dorsheimer L, Rieger MA, Dimmeler S (2020). Clonal haematopoiesis in patients with degenerative aortic valve stenosis undergoing transcatheter aortic valve implantation. Eur Heart J.

[18] Jaiswal S, Natarajan P, Silver AJ, Gibson CJ, Bick AG, Shvartz E (2017). Clonal hematopoiesis and risk of atherosclerotic cardiovascular disease. N. Engl J Med.

[19] Cremer S, Kirschbaum K, Berkowitsch A, John D, Kiefer K, Dorsheimer L (2020). Multiple somatic mutations for clonal hematopoiesis are associated with increased mortality in patients with chronic heart failure. Circ Genom Precis Med.

[20] Coombs CC, Zehir A, Devlin SM, Kishtagari A, Syed A, Jonsson P (2017). Therapy-related clonal hematopoiesis in patients with non-hematologic cancers is common and associated with adverse clinical outcomes. Cell Stem Cell.

[21] Bick AG, Weinstock JS, Nandakumar SK, Fulco CP, Leventhal MJ, Bao EL, et al. Inherited causes of clonal hematopoiesis of indeterminate potential in TOPMed whole genomes. bioRxiv. 2019. 10.1101/782748.

[22] Zink F, Stacey SN, Norddahl GL, Frigge ML, Magnusson OT, Jonsdottir I (2017). Clonal hematopoiesis, with and without candidate driver mutations, is common in the elderly. Blood.

[23] Hsu JI, Dayaram T, Tovy A, De Braekeleer E, Jeong M, Wang F (2018). PPM1D mutations drive clonal hematopoiesis in response to cytotoxic chemotherapy. Cell Stem Cell.

[24] Haferlach T, Nagata Y, Grossmann V, Okuno Y, Bacher U, Nagae G (2014). Landscape of genetic lesions in 944 patients with myelodysplastic syndromes. Leukemia.

[25] Ley TJ, Ding L, Walter MJ, McLellan MD, Lamprecht T, Larson DE (2010). DNMT3A mutations in acute myeloid leukemia. N. Engl J Med.

[26] Chou WC, Huang HH, Hou HA, Chen CY, Tang JL, Yao M (2010). Distinct clinical and biological features of de novo acute myeloid leukemia with additional sex comb-like 1 (ASXL1) mutations. Blood.

[27] Delhommeau F, Dupont S, Valle VD, James C, Trannoy S, Massé A (2009). Mutation in TET2 in myeloid cancers. N Engl J Med.

[28] Weissmann S, Alpermann T, Grossmann V, Kowarsch A, Nadarajah N, Eder C (2012). Landscape of TET2 mutations in acute myeloid leukemia. Leukemia.

[29] Holz-Schietinger C, Matje DM, Reich NO (2012). Mutations in DNA methyltransferase (DNMT3A) observed in acute myeloid leukemia patients disrupt processive methylation. J Biol Chem.

[30] Chuman Y, Kurihashi W, Mizukami Y, Nashimoto T, Yagi H, Sakaguchi K (2009). PPM1D430, a novel alternative splicing variant of the human PPM1D, can dephosphorylate p53 and exhibits specific tissue expression. J Biochem.

[31] Kleiblova P, Shaltiel IA, Benada J, Ševčík J, Pecháčková S, Pohlreich P (2013). Gain-of-function mutations of PPM1D/Wip1 impair the p53-dependent G1 checkpoint. J Cell Biol.

[32] Kahn JD, Miller PG, Silver AJ, Sellar RS, Bhatt S, Gibson C (2018). PPM1D-truncating mutations confer resistance to chemotherapy and sensitivity to PPM1D inhibition in hematopoietic cells. Blood.

[33] Bulavin DV, Demidov ON, Saito S, Kauraniemi P, Phillips C, Amundson SA (2002). Amplification of PPM1D in human tumors abrogates p53 tumor-suppressor activity. Nat Genet.

[34] Li J, Yang Y, Peng Y, Austin RJ, van Eyndhoven WG, Nguyen KCQ (2002). Oncogenic properties of PPM1D located within a breast cancer amplification epicenter at 17q23. Nat Genet.

[35] Kochetkova EU, Grigorash BBDO (2019). Sensitivity of cells with different levels of expression of the ppm1d gene to the action of the classical combination of chemicals for the treatment of column cancer. Cell Tissue Biol.

[36] Arends CM, Galan-Sousa J, Hoyer K, Chan W, Jäger M, Yoshida K (2018). Hematopoietic lineage distribution and evolutionary dynamics of clonal hematopoiesis. Leukemia.

[37] Buscarlet M, Provost S, Zada YF, Bourgoin V, Mollica L, Dubé MP (2018). Lineage restriction analyses in CHIP indicate myeloid bias for TET2 and multipotent stem cell origin for DNMT3A. Blood.

[38] Hartmann L, Hecker JS, Rothenberg-Thurley M, Rivière J, Jentzsch M, Ksienzyk B (2022). Compartment-specific mutational landscape of clonal hematopoiesis. Leukemia.

[39] Franceschi C, Zaikin A, Gordleeva S, Ivanchenko M, Bonifazi F, Storci G (2018). Inflammaging 2018: an update and a model. Semin Immunol.

[40] Choi J, Nannenga B, Demidov ON, Bulavin DV, Cooney A, Brayton C (2002). Mice deficient for the wild-type p53-induced phosphatase gene (Wip1) exhibit defects in reproductive organs, immune function, and cell cycle control. Mol Cell Biol.

[41] Hou HA, Chou WC, Kuo YY, Liu CY, Lin LI, Tseng MH (2015). TP53 mutations in de novo acute myeloid leukemia patients: longitudinal follow-ups show the mutation is stable during disease evolution. Blood Cancer J.

[42] Uyanik B, Grigorash BB, Goloudina AR, Demidov ON (2017). DNA damage-induced phosphatase Wip1 in regulation of hematopoiesis, immune system and inflammation. Cell Death Discov.

[43] Lu X, Nguyen TA, Moon SH, Darlington Y, Sommer M, Donehower LA (2008). The type 2C phosphatase Wip1: an oncogenic regulator of tumor suppressor and DNA damage response pathways. Cancer Metast Rev.

[44] Lowe J, Cha H, Lee MO, Mazur SJ, Appella E, Fornace AJ (2012). Regulation of the Wip1 phosphatase and its effects on the stress response. Front Biosci.

[45] Chen Z, Yi W, Morita Y, Wang H, Cong Y, Liu JP (2015). Wip1 deficiency impairs haematopoietic stem cell function via p53 and mTORC1 pathways. Nat Commun.

[46] Demidov ON, Kek C, Shreeram S, Timofeev O, Fornace AJ, Appella E (2007). The role of the MKK6/p38 MAPK pathway in Wip1-dependent regulation of ErbB2-driven mammary gland tumorigenesis. Oncogene.

[47] Demidov ON, Timofeev O, Lwin HNY, Kek C, Appella E, Bulavin DV (2007). Wip1 phosphatase regulates p53-dependent apoptosis of stem cells and tumorigenesis in the mouse intestine. Cell Stem Cell.

[48] Lee JS, Lee MO, Moon BH, Shim SH, Fornace AJ, Cha HJ (2009). Senescent growth arrest in mesenchymal stem cells is bypassed by Wip1-mediated downregulation of intrinsic stress signaling pathways. Stem Cells.

[49] Zhang L, Liu L, He Z, Li G, Liu J, Song Z (2015). Inhibition of wild-type p53-induced phosphatase 1 promotes liver regeneration in mice by direct activation of mammalian target of rapamycin. Hepatology.

[50] Le Guezennec X, Brichkina A, Huang YF, Kostromina E, Han W, Bulavin DV (2012). Wip1-dependent regulation of autophagy, obesity, and atherosclerosis. Cell Metab.

[51] Fontana MC, Nanni J, Ghelli Luserna di Rorà A, Petracci E, Padella A, Ghetti M (2021). Pharmacological inhibition of WIP1 sensitizes acute myeloid leukemia cells to the MDM2 inhibitor nutlin-3a. Biomedicines.

[52] Tang Y, Pan B, Zhou X, Xiong K, Gao Q, Huang L (2017). Wip1-dependent modulation of macrophage migration and phagocytosis. Redox Biol.

[53] Yin S, Wang P, Yang L, Liu Y, Wang Y, Liu M (2016). Wip1 suppresses ovarian cancer metastasis through the ATM/AKT/Snail mediated signaling. Oncotarget.

[54] Buss MC, Remke M, Lee J, Gandhi K, Schniederjan MJ, Kool M (2015). The WIP1 oncogene promotes progression and invasion of aggressive medulloblastoma variants. Oncogene.

[55] Schito ML, Demidov ON, Saito S, Ashwell JD, Appella E (2006). Wip1 phosphatase-deficient mice exhibit defective T cell maturation due to sustained p53 activation. J Immunol.

[56] Yi W, Hu X, Chen Z, Liu L, Tian Y, Chen H (2015). Phosphatase Wip1 controls antigen-independent B-cell development in a p53-dependent manner. Blood.

[57] Sun L, Li H, Luo H, Zhang L, Hu X, Yang T (2013). Phosphatase Wip1 is essential for the maturation and homeostasis of medullary thymic epithelial cells in mice. J Immunol.

[58] Hu X, Wang P, Du J, Yang F, Tian Y, Shen X (2016). Phosphatase Wip1 masters IL-17–producing neutrophil-mediated colitis in mice. Inflamm Bowel Dis.

[59] Tan X, Zhang J, Jin W, Li L, Xu W, Zheng H (2013). Wip1 phosphatase involved in lipopolysaccharide-induced neuroinflammation. J Mol Neurosci.

[60] Zhong H, Cui L, Xu F, Chen L, Jiang L, Huang H (2016). Up-regulation of Wip1 involves in neuroinflammation of retinal astrocytes after optic nerve crush via NF-κB signaling pathway. Inflamm Res.

[61] Liu G, Hu X, Sun B, Yang T, Shi J, Zhang L (2013). Phosphatase Wip1 negatively regulates neutrophil development through p38 MAPK-STAT1. Blood.

[62] Bick AG, Pirruccello JP, Griffin GK, Gupta N, Gabriel S, Saleheen D (2020). Genetic interleukin 6 signaling deficiency attenuates cardiovascular risk in clonal hematopoiesis. Circulation.

[63] Hecker JS, Hartmann L, Rivière J, Buck MC, van der Garde M, Rothenberg-Thurley M (2021). CHIP & HIPs: clonal hematopoiesis is common in hip arthroplasty patients and associates with autoimmune disease. Blood.

[64] Dorsheimer L, Assmus B, Rasper T, Ortmann CA, Ecke A, Abou-El-Ardat K (2019). Association of mutations contributing to clonal hematopoiesis with prognosis in chronic ischemic heart failure. JAMA Cardiol.

[65] Jan M, Ebert BL, Jaiswal S (2017). Clonal hematopoiesis. Semin Hematol.

[66] Shlush LI (2018). Age-related clonal hematopoiesis. Blood.

[67] Verovskaya E, Broekhuis MJC, Zwart E, Ritsema M, van Os R, de Haan G (2013). Heterogeneity of young and aged murine hematopoietic stem cells revealed by quantitative clonal analysis using cellular barcoding. Blood.

[68] van den Akker EB, Pitts SJ, Deelen J, Moed MH, Potluri S, van Rooij J (2016). Uncompromised 10-year survival of oldest old carrying somatic mutations in DNMT3A and TET2. Blood.

[69] van Zeventer IA, Salzbrunn JB, de Graaf AO, van der Reijden BA, Boezen HM, Vonk JM (2021). Prevalence, predictors, and outcomes of clonal hematopoiesis in individuals aged ≥80 years. Blood Adv.

[70] Antoni BG, Miriam DD, Álvaro HV, David VA, Jorge de la B, A. PFD (2021). Clonal hematopoiesis and risk of progression of heart failure with reduced left ventricular ejection fraction. J Am Coll Cardiol.

[71] Jaiswal S, Libby P (2020). Clonal haematopoiesis: connecting ageing and inflammation in cardiovascular disease. Nat Rev Cardiol.

[72] Cook EK, Izukawa T, Young S, Rosen G, Jamali M, Zhang L (2019). Comorbid and inflammatory characteristics of genetic subtypes of clonal hematopoiesis. Blood Adv.

[73] Fuster JJ, MacLauchlan S, Zuriaga MA, Polackal MN, Ostriker AC, Chakraborty R (2017). Clonal hematopoiesis associated with TET2 deficiency accelerates atherosclerosis development in mice. Science.

[74] Heyde A, Rohde D, McAlpine CS, Zhang S, Hoyer FF, Gerold JM (2021). Increased stem cell proliferation in atherosclerosis accelerates clonal hematopoiesis. Cell.

[75] Lusis AJ (2021). A vicious cycle in atherosclerosis. Cell.

[76] Grigorash BB, Uyanik B, Kochetkova EY, Goloudina AR, Demidov ON (2017). Wip1 inhibition leads to severe pro-inflammatory phenotype in skin in response to chemical irritation. J Dermatol Sci.

[77] Goloudina AR, Kochetkova EY, Pospelova TV, Demidov ON (2016). Wip1 phosphatase: between p53 and MAPK kinases pathways. Oncotarget.

[78] Ruark E, Snape K, Humburg P, Loveday C, Bajrami I, Brough R (2013). Mosaic PPM1D mutations are associated with predisposition to breast and ovarian cancer. Nature.

[79] Bolton KL, Koh Y, Foote MB, Im H, Jee J, Sun CH, et al. Clonal hematopoiesis is associated with risk of severe Covid-19. medRxiv. 2020;11.25.20233163. 10.1101/2020.11.25.20233163. 10.1038/s41467-021-26138-6 PMC8514469 34645798

[80] Dharan NJ, Yeh P, Bloch M, Yeung MM, Baker D, Guinto J (2021). HIV is associated with an increased risk of age-related clonal hematopoiesis among older adults. Nat Med.

[81] Duployez N, Demonchy J, Berthon C, Goutay J, Caplan M, Moreau AS (2020). Clinico-biological features and clonal hematopoiesis in patients with severe covid-19. Cancers (Basel).

[82] Petzer V, Schwendinger S, Haschka D, Vogi V, Tymoszuk P, Burkert F (2021). Clonal hematopoiesis in patients with COVID-19 is stable and not linked to an aggravated clinical course. Am J Hematol.

[83] Hameister E, Stolz SM, Fuhrer Y, Thienemann F, Schaer DJ, Nemeth J (2020). Clonal hematopoiesis in hospitalized elderly patients with COVID-19. Hemasphere.

[84] Busque L, Sun M, Buscarlet M, Ayachi S, Feroz Zada Y, Provost S (2020). High-sensitivity C-reactive protein is associated with clonal hematopoiesis of indeterminate potential. Blood Adv.

[85] Han H, Ma Q, Li C, Liu R, Zhao L, Wang W (2020). Profiling serum cytokines in COVID-19 patients reveals IL-6 and IL-10 are disease severity predictors. Emerg Microbes Infect.

[86] Wang G, Wu C, Zhang Q, Wu F, Yu B, Lv J (2020). C-reactive protein level may predict the risk of COVID-19 aggravation. Open Forum Infect Dis.

[87] Chan HT, Nagayama S, Chin YM, Otaki M, Hayashi R, Kiyotani K (2020). Clinical significance of clonal hematopoiesis in the interpretation of blood liquid biopsy. Mol Oncol.

[88] Razavi P, Li BT, Brown DN, Jung B, Hubbell E, Shen R (2019). High-intensity sequencing reveals the sources of plasma circulating cell-free DNA variants. Nat Med.

[89] Okamura R, Piccioni DE, Boichard A, Lee S, Jimenez RE, Sicklick JK (2021). High prevalence of clonal hematopoiesis-type genomic abnormalities in cell-free DNA in invasive gliomas after treatment. Int J Cancer.

[90] Suehara Y, Sakata-Yanagimoto M, Hattori K, Kusakabe M, Nanmoku T, Sato T (2019). Mutations found in cell-free DNAs of patients with malignant lymphoma at remission can derive from clonal hematopoiesis. Cancer Sci.

[91] Okano M, Xie S, Li E (1998). Cloning and characterization of a family of novel mammalian DNA (cytosine-5) methyltransferases. Nat Genet.

[92] Kaneda M, Okano M, Hata K, Sado T, Tsujimoto H, Li E (2004). Essential role for de novo DNA methyltransferase Dnmt3a in paternal and maternal imprinting. Nature.

[93] Ley TJ, Miller C, Ding L, Raphael BJ, Mungall AJ, Robertson A (2013). Genomic and epigenomic landscapes of adult de novo acute myeloid leukemia. N Engl J Med.

[94] Yuan XQ, Peng L, Zeng WJ, Jiang BY, Li GC, Chen XP (2016). DNMT3A R882 mutations predict a poor prognosis in AML: a meta-analysis from 4474 patients. Medicine.

[95] Thol F, Winschel C, Lüdeking A, Yun H, Friesen I, Damm F (2011). Rare occurrence of DNMT3A mutations in myelodysplastic syndromes. Haematologica.

[96] Walter MJ, Ding L, Shen D, Shao J, Grillot M, Fulton R (2012). Recurrent DNMT3A mutations in patients with myelodysplastic syndromes. Leukemia.

[97] Brecqueville M, Cervera N, Gelsi-Boyer V, Murati A, Adélaïde J, Chaffanet M (2011). Rare mutations in DNMT3A in myeloproliferative neoplasms and myelodysplastic syndromes. Blood Cancer J.

[98] Roller A, Grossmann V, Bacher U, Poetzinger F, Weissmann S, Nadarajah N (2013). Landmark analysis of DNMT3A mutations in hematological malignancies. Leukemia.

[99] Chesnais V, Renneville A, Toma A, Lambert J, Passet M, Dumont F (2016). Effect of lenalidomide treatment on clonal architecture of myelodysplastic syndromes without 5q deletion. Blood.

[100] Ribeiro AFT, Pratcorona M, Erpelinck-Verschueren C, Rockova V, Sanders M, Abbas S (2012). Mutant DNMT3A: a marker of poor prognosis in acute myeloid leukemia. Blood.

[101] Sano S, Oshima K, Wang Y, MacLauchlan S, Katanasaka Y, Sano M (2018). Tet2-mediated clonal hematopoiesis accelerates heart failure through a mechanism involving the IL-1β/NLRP3 inflammasome. J Am Coll Cardiol.

[102] Sano S, Oshima K, Wang Y, Katanasaka Y, Sano M, Walsh K (2018). CRISPR-mediated gene editing to assess the roles of Tet2 and Dnmt3a in clonal hematopoiesis and cardiovascular disease. Circ Res.

[103] Jan M, Snyder TM, Corces-Zimmerman MR, Vyas P, Weissman IL, Quake SR (2012). Clonal evolution of preleukemic hematopoietic stem cells precedes human acute myeloid leukemia. Sci Transl Med.

[104] Link DC, Walter MJ (2016). ‘CHIP’ping away at clonal hematopoiesis. Leukemia.

[105] Ito S, Shen L, Dai Q, Wu SC, Collins LB, Swenberg JA (2011). Tet proteins can convert 5-methylcytosine to 5-formylcytosine and 5-carboxylcytosine. Science (1979).

[106] Ko M, Huang Y, Jankowska AM, Pape UJ, Tahiliani M, Bandukwala HS (2011). Impaired hydroxylation of 5-methylcytosine in myeloid cancers with mutant TET2. Nature.

[107] Moran-Crusio K, Reavie L, Shih A, Abdel-Wahab O, Ndiaye-Lobry D, Lobry C (2011). Tet2 loss leads to increased hematopoietic stem cell self-renewal and myeloid transformation. Cancer Cell.

[108] Rasmussen KD, Jia G, Johansen JV, Pedersen MT, Rapin N, Bagger FO (2015). Loss of TET2 in hematopoietic cells leads to DNA hypermethylation of active enhancers and induction of leukemogenesis. Genes Dev.

[109] Langemeijer SMC, Kuiper RP, Berends M, Knops R, Aslanyan MG, Massop M (2009). Acquired mutations in TET2 are common in myelodysplastic syndromes. Nat Genet.

[110] Bejar R, Stevenson K, Abdel-Wahab O, Galili N, Nilsson B, Garcia-Manero G (2011). Clinical effect of point mutations in myelodysplastic syndromes. N. Engl J Med.

[111] Papaemmanuil E, Gerstung M, Malcovati L, Tauro S, Gundem G, Van Loo P (2013). Clinical and biological implications of driver mutations in myelodysplastic syndromes. Blood.

[112] Wang R, Gao X, Yu L (2019). The prognostic impact of tet oncogene family member 2 mutations in patients with acute myeloid leukemia: a systematic-review and meta-analysis. BMC Cancer.

[113] Abdel-Wahab O, Mullally A, Hedvat C, Garcia-Manero G, Patel J, Wadleigh M (2009). Genetic characterization of TET1, TET2, and TET3 alterations in myeloid malignancies. Blood.

[114] Lin PH, Li HY, Fan SC, Yuan TH, Chen M, Hsu YH (2017). A targeted next-generation sequencing in the molecular risk stratification of adult acute myeloid leukemia: implications for clinical practice. Cancer Med.

[115] Abdel-Wahab O, Adli M, LaFave LM, Gao J, Hricik T, Shih AH (2012). ASXL1 mutations promote myeloid transformation through loss of PRC2-mediated gene repression. Cancer Cell.

[116] Asada S, Kitamura T (2019). Aberrant histone modifications induced by mutant ASXL1 in myeloid neoplasms. Int J Hematol.

[117] Asada S, Goyama S, Inoue D, Shikata S, Takeda R, Fukushima T (2018). Mutant ASXL1 cooperates with BAP1 to promote myeloid leukaemogenesis. Nat Commun.

[118] Balasubramani A, Larjo A, Bassein JA, Chang X, Hastie RB, Togher SM (2015). Cancer-associated ASXL1 mutations may act as gain-of-function mutations of the ASXL1–BAP1 complex. Nat Commun.

[119] Fujino T, Goyama S, Sugiura Y, Inoue D, Asada S, Yamasaki S (2021). Mutant ASXL1 induces age-related expansion of phenotypic hematopoietic stem cells through activation of Akt/mTOR pathway. Nat Commun.

[120] Inoue D, Matsumoto M, Nagase R, Saika M, Fujino T, Nakayama KI (2016). Truncation mutants of ASXL1 observed in myeloid malignancies are expressed at detectable protein levels. Exp Hematol.

[121] Schnittger S, Eder C, Jeromin S, Alpermann T, Fasan A, Grossmann V (2013). ASXL1 exon 12 mutations are frequent in AML with intermediate risk karyotype and are independently associated with an adverse outcome. Leukemia.

[122] Pratcorona M, Abbas S, Sanders MA, Koenders JE, Kavelaars FG, Erpelinck-Verschueren CAJ (2012). Acquired mutations in ASXL1 in acute myeloid leukemia: prevalence and prognostic value. Haematologica.

[123] Asada S, Fujino T, Goyama S, Kitamura T (2019). The role of ASXL1 in hematopoiesis and myeloid malignancies. Cell Mol Life Sci.

[124] Fiscella M, Zhang HL, Fan S, Sakaguchi K, Shen SF, Mercer W (1997). EWip1, a novel human protein phosphatase that is induced in response to ionising radiation in a p53-dependent manner. Proc Natl Acad Sci USA.

[125] Barford D, Das AK, Egloff MP (1998). The structure and mechanism of protein phosphatases: insights into catalysis and regulation. Annu Rev Biophys Biomol Struct.

[126] Lindsley RC, Saber W, Mar BG, Redd R, Wang T, Haagenson MD (2017). Prognostic mutations in myelodysplastic syndrome after stem-cell transplantation. N Engl J Med.

[127] Kartal-Kaess M, Bochtler T, Kraft B, Kirsch M, Maier B, Stoelzel F (2018). PPM1D mutations are rare in de novo and therapy-related acute myeloid leukemia. Blood.

[128] Levine AJ, Oren M (2009). The first 30 years of p53: growing ever more complex. Nat Rev Cancer.

[129] Chen S, Wang Q, Yu H, Capitano ML, Vemula S, Nabinger SC (2019). Mutant p53 drives clonal hematopoiesis through modulating epigenetic pathway. Nat Commun.

[130] Kulasekararaj AG, Smith AE, Mian SA, Mohamedali AM, Krishnamurthy P, Lea NC (2013). TP53 mutations in myelodysplastic syndrome are strongly correlated with aberrations of chromosome 5, and correlate with adverse prognosis. Br J Haematol.

[131] Olivier M, Hollstein M, Hainaut P (2010). TP53 mutations in human cancers: origins, consequences, and clinical use. Cold Spring Harb Perspect Biol.

[132] Kadia TM, Jain P, Ravandi F, Garcia-Manero G, Andreef M, Takahashi K (2016). TP53 mutations in newly diagnosed acute myeloid leukemia: clinicomolecular characteristics, response to therapy, and outcomes. Cancer.

[133] Metzeler KH, Herold T, Rothenberg-Thurley M, Amler S, Sauerland MC, Görlich D (2016). Spectrum and prognostic relevance of driver gene mutations in acute myeloid leukemia. Blood.

[134] Wong TN, Ramsingh G, Young AL, Miller CA, Touma W, Welch JS (2015). Role of TP53 mutations in the origin and evolution of therapy-related acute myeloid leukaemia. Nature.

[135] Nelson ME, Steensma DP (2006). JAK2 V617F in myeloid disorders: What do we know now, and where are we headed?. Leuk Lymphoma.

[136] James C, Ugo V, Le Couédic JP, Staerk J, Delhommeau F, Lacout C (2005). A unique clonal JAK2 mutation leading to constitutive signalling causes polycythaemia vera. Nature.

[137] Nielsen C, Birgens HS, Nordestgaard BG, Bojesen SE (2013). Diagnostic value of JAK2 V617F somatic mutation for myeloproliferative cancer in 49 488 individuals from the general population. Br J Haematol.

[138] Nielsen C, Birgens HS, Nordestgaard BG, Kjaer L, Bojesen SE (2011). The JAK2 V617F somatic mutation, mortality and cancer risk in the general population. Haematologica.

[139] Sidon P, El Housni H, Dessars B, Heimann P (2006). The JAK2V617F mutation is detectable at very low level in peripheral blood of healthy donors. Leukemia.

[140] Steensma DP, Dewald GW, Lasho TL, Powell HL, McClure RF, Levine RL (2005). The JAK2 V617F activating tyrosine kinase mutation is an infrequent event in both ‘atypical’ myeloproliferative disorders and myelodysplastic syndromes. Blood.

[141] Jelinek J, Oki Y, Gharibyan V, Bueso-Ramos C, Prchal JT, Verstovsek S (2005). JAK2 mutation 1849G>T is rare in acute leukemias but can be found in CMML, Philadelphia chromosome-negative CML, and megakaryocytic leukemia. Blood.

[142] Lee JW, Kim YG, Soung YH, Han KJ, Kim SY, Rhim HS (2006). The JAK2 V617F mutation in de novo acute myelogenous leukemias. Oncogene.

[143] Steensma DP, McClure RF, Karp JE, Tefferi A, Lasho TL, Powell HL (2006). JAK2 V617F is a rare finding in de novo acute myeloid leukemia, but STAT3 activation is common and remains unexplained. Leukemia.

[144] Swaminathan S, Madkaikar M, Ghosh K, Vundinti BR, Kerketta L, Gupta M (2010). Novel immunophenotypic and morphologic presentation in acute myeloid leukemia (AML) with JAK2 V617F mutation. Eur J Haematol.

[145] Illmer T, Schaich M, Ehninger G, Thiede C (2007). Tyrosine kinase mutations of JAK2 are rare events in AML but influence prognosis of patients with CBF-leukemias. Haematologica.

[146] Balatzenko G, Spassov B, Georgieva Y, Hrischev V, Guenova M (2015). Low incidence of V617FJAK2 mutation in acute myeloid leukemia and myelodysplastic syndromes. Blood.

[147] Hinds DA, Barnholt KE, Mesa RA, Kiefer AK, Do CB, Eriksson N (2016). Germ line variants predispose to both JAK2 V617F clonal hematopoiesis and myeloproliferative neoplasms. Blood.

[148] Wolach O, Sellar RS, Martinod K, Cherpokova D, McConkey M, Chappell RJ (2018). Increased neutrophil extracellular trap formation promotes thrombosis in myeloproliferative neoplasms. Sci Transl Med.

[149] Wang W, Liu W, Fidler T, Wang Y, Tang Y, Woods B (2018). Macrophage inflammation, erythrophagocytosis, and accelerated atherosclerosis in Jak2 (V617F) mice. Circ Res.

